# Association between exposure to secondhand smoking at home and tooth loss in Japan: A cross-sectional analysis of data from the 2016 National Health and Nutrition Survey

**DOI:** 10.18332/tid/143177

**Published:** 2021-12-09

**Authors:** Yuko Inoue, Takashi Zaitsu, Oshiro Akiko, Miho Ishimaru, Kento Taira, Hideto Takahashi, Jun Aida, Nanako Tamiya

**Affiliations:** 1 Department of Oral Health Promotion, Graduate School of Medical and Dental Sciences, Tokyo Medical and Dental University, Tokyo, Japan; 2 Department of Health Services Research, School of Medicine, University of Tsukuba, Tsukuba, Japan; 3 National Institute of Public Health, Saitama, Japan

**Keywords:** oral health, secondhand smoking, tooth loss, Japan

## Abstract

**INTRODUCTION:**

Tooth loss affects oral health and physical and social functions. With widespread population aging, its prevalence is increasing. Secondhand smoking is a risk factor for oral diseases; however, in Japan, there are currently no regulations restricting exposure to secondhand smoke at home. This cross-sectional study examined the association between secondhand smoking at home and tooth loss among Japanese adults.

**METHODS:**

The study examined secondary data from the 2016 National Health and Nutrition Survey, Japan. The self-reported responses of 18812 non-smokers aged ≥20 years were analyzed. The association between exposure to secondhand smoke at home and number of teeth was examined through multiple linear regression with multiple imputation. To obtain a more normal distribution, logarithmic transformation was applied to the number of teeth that deviated substantially from a normal distribution. Adjustments were made for sex, age, occupation, household size, dental check-up within the past year, and exposure to secondhand smoke outside the home.

**RESULTS:**

Overall, 8.4% of the respondents were exposed to secondhand smoking at home almost every day; this percentage was larger among younger respondents, who also had more teeth than the older respondents. Although the univariate linear regression did not show a positive association between exposure to secondhand smoke and tooth loss, the multivariate-adjusted analysis revealed that respondents who were exposed to secondhand smoke at home almost every day had fewer teeth (β= -0.04; 95% CI: -0.07 – -0.01).

**CONCLUSIONS:**

The present results suggest that exposure to secondhand smoke at home increases the risk of tooth loss.

## INTRODUCTION

The oral cavity has a variety of physical and social functions, such as eating, chewing, and speaking^1,2^. Therefore, tooth loss can significantly affect the quality of life^3^ and general health conditions^4,5^. Tooth loss is a consequence of oral disease, and the primary causes include dental caries and periodontal disease^6,7^, Further, the burden of total tooth loss is greater than that of caries or periodontal disease^8^. Recently, the global rate of tooth loss has increased as a result of widespread population aging^9^, therefore maintaining oral health in aging societies has become a major health concern.

It is well known that smoking has a profound exacerbating effect on the major causes of tooth loss^10-12^. Not just active, but secondhand smoking also has harmful effects on oral health, causing tooth loss^13,14^, severe periodontitis^15^, dental caries^16^, and gingival pigmentation^17^. Exposure to secondhand smoke results in the involuntary inhalation of the same harmful substances (nicotine, tar, carbon monoxide, etc.) that active smokers inhale^18^.

While in many countries, smoking is restricted or banned in public places to reduce public exposure to secondhand smoke, in Japan there is no law restricting the practice at home. Further, dentistry-based studies on the harmful effects of secondhand smoking at home have largely examined only children^19^ and pregnant women^14^; few studies have focused on tooth loss in adults. Therefore, the present study aimed to examine the association between exposure to secondhand smoke at home and tooth loss. We hypothesized that exposure to secondhand smoke at home is associated with tooth loss in non-smokers.

## METHODS

### Data

Our cross-sectional study represents a secondary analysis of data collected for the 2016 National Health and Nutrition Survey, Japan (NHNS)^20^ and was conducted with permission from the Japanese Ministry of Health, Labor and Welfare. The NHNS is a nationwide survey using a stratified random sampling method conducted annually by public health centers with jurisdiction over the survey area under the supervision of the Ministry of Health, Labor and Welfare, with the aim of clarifying the physical condition, nutritional intake, and lifestyle of the Japanese people^21^. The survey was conducted from October to November 2016. In total, 10745 of 24187 eligible households (44.4%) and 26225 people participated.

As we had aimed to determine the health effects of secondhand smoking on adults, we excluded current and past smokers and people aged <20 years from our analysis. Consequently, we analyzed data for 18812 respondents.

The NHNS was conducted in accordance with the Declaration of Helsinki after obtaining verbal informed consent from all respondents. Under the Statistics Act, the Ministry of Health, Labor and Welfare anonymized individual-level data obtained from the NHNS and provided the datasets for this study. In accordance with the Ethical Guidelines of Epidemiological Research in Japan, this secondary analysis of anonymous data was exempted from ethical review.

### Dependent variable

Respondents’ number of teeth was assessed by the question: ‘How many teeth do you have, excluding wisdom teeth, dentures, dental bridges, and implants?’.

### Independent variables

As variables, we used exposure to secondhand smoke at home, age, occupation, household size, whether the respondent had a dental check-up within the past year, and exposure to secondhand smoke outside the home (workplaces, schools, restaurants, amusement parks, government buildings, hospitals, public transportation, streets, environments with children). Choices of questions with fewer responses were integrated. Frequency of secondhand smoke exposure was measured using the following question: ‘Have you been exposed to secondhand smoking in the past month?’. The possible responses were: ‘almost every day’, ‘several times a week’, ‘once a week’, ‘once a month’, ‘never’, and ‘did not go out’. These were grouped into three categories: 1) ‘several times a week’, ‘once a week’, and ‘once a month’ were combined into ‘several times a week to once a month’; 2) ‘never’ and ‘did not go out’ were combined into ‘never’; and 3) ‘almost every day’. In the sensitivity analysis, we included the original five categories variable of secondhand smoke exposure and built the same model as the main analyses. Age was divided into four groups: 20–39, 40–59, 60–79, and ≥80 years. Occupation was divided into six groups: upper white-collar, lower white-collar, blue-collar, homemaker, student, and other. Household size (persons) was divided into four categories: 1, 2, 3, and ≥4. Dental check-up was divided into two categories: those who did and did not have a dental check-up within the past year.

### Statistical analysis

The mean number of teeth was calculated for each variable. Then, the association between exposure to secondhand smoke at home and the number of teeth was examined using multiple linear regression. Before performing the regression, to obtain a more normal distribution, logarithmic transformation was applied to the number of teeth, if their distribution deviated substantially from a normal distribution. Adjustments were made for sex, age, occupation, household size, dental check-up within the past year, and exposure to secondhand smoke outside the home. For missing values, we used multiple imputation (MI). We replaced each missing value with a substitute set of plausible values by using MI with chained equations to create 10 complete datasets^22^. The following variables were used to create these complete datasets: age, sex, occupation, dental check-up within the past year, household size, and exposure to secondhand smoke at home and outside the home. All data analyses were conducted using STATA^®^ 15.1 (Stata Corporation, College Station, TX, USA). Statistical significance was set at p<0.05.

## RESULTS

Of the 26225 NHNSJ respondents, analysis was performed on the data of 18812 (71.7%) non-smokers. Among these, 65.5% were women, and the mean age was 58.8 (SD: 18.5) for men and 59.0 (SD: 18.1) for women.

Table 1 shows the participants’ demographic characteristics (after applying MI) in terms of their exposure to secondhand smoke at home. The prevalence in the sample of exposure to secondhand smoke at home ‘almost every day’, ‘several times a week to once a month’, and ‘never’ was 8.4%, 6.6%, and 85.0%, respectively. A large sex-based difference was observed; the corresponding prevalence rates were 3.6%, 4.0%, and 92.4% for men and 10.9%, 7.9%, and 81.2% for women, respectively. Women, lower white-collar workers, blue-collar workers, homemakers, those with larger households, and those who were exposed to secondhand smoke outside the home almost every day tended to be more exposed to secondhand smoking at home. For those who reported exposure to secondhand smoke ‘almost every day’, ‘several times a week to once a month’, and ‘never’, the mean number of teeth was 21.7 (SD: 8.8), 22.6 (SD: 8.3), and 21.2 (SD: 9.0), respectively. The mean number of teeth decreased with age with no difference between men (21.4, SD: 9.0) and women (21.4, SD: 8.9) in this regard.

**Table 1:** Characteristics of the study population and frequency of secondhand smoking exposure in the home, after performing multiple imputation (N=18812)

*Characteristics*	*Total*	*Number of teeth*	*Almost every day*	*Several times a week to once a month*	*Never*
	*n (%)*	*Mean ± SD*	*%*	*%*	*%*
**Total**, n (%)	18812 (100)		1580 (8.4)	1234 (6.6)	15998 (85.0)
**Sex**					
Men	6484 (34.5)	21.4 ± 9.0	3.6	4.0	92.4
Women	12328 (65.5)	21.4 ± 8.9	10.9	7.9	81.2
**Age** (years)					
20–39	3356 (17.8)	27.6 ± 1.8	10.1	10.8	79.1
40–59	5215 (27.7)	25.8 ± 4.3	9.8	6.7	83.6
60–79	7820 (41.6)	19.1 ± 8.7	7.7	5.5	86.9
≥80	2421 (12.9)	10.4 ± 9.9	5.5	4.0	90.5
**Occupation**					
Upper white-collar	2836 (15.1)	25.7 ± 4.9	6.7	6.3	87.0
Lower white-collar	4612 (24.5)	24.6 ± 6.2	10.0	7.5	82.5
Blue-collar	2613 (13.9)	21.8 ± 8.4	9.2	7.5	83.2
Homemaker	4792 (25.5)	20.1 ± 9.0	9.3	6.7	84.0
Student	236 (1.3)	26.4 ± 5.6	9.3	10.9	79.8
Other	3724 (19.8)	15.0 ± 10.6	5.9	4.5	89.5
**Household size** (persons)					
1	2481 (13.2)	18.3 ± 9.9	3.0	5.6	91.3
2	6717 (35.7)	20.3 ± 9.0	6.9	4.6	88.6
3	4260 (22.7)	22.2 ± 8.7	10.6	6.9	82.5
≥4	5354 (28.5)	23.5 ± 8.0	11.1	9.2	79.7
**Dental check-up within the past year**					
Yes	10107 (53.7)	22.2 ± 7.8	7.8	6.3	85.9
No	8705 (46.3)	20.4 ± 10.1	9.1	6.8	84.0
**Frequency of exposure to secondhand smoke outside the home**					
Almost every day	1376 (7.3)	23.7 ± 7.2	17.5	4.8	77.6
Several times a week to once a month	7626 (40.5)	23.6 ± 7.3	7.7	7.7	84.6
Never	9810 (52.2)	19.4 ± 9.8	7.7	5.9	86.4
**Number of teeth**, mean ± SD			21.7 ± 8.8	22.6 ± 8.3	21.2 ± 9.0

SD: standard deviation.

Table 2 shows all the participants in the first survey, except those who did not answer the question about smoking status. A higher percentage of respondents aged 30–39, 40–49, and 50–59 years reported smoking every day (25.9%, 25.5%, and 22.1%, respectively) compared to other age groups.

**Table 2:** Smoking status (%) by age group*

	*Age (years)*							
	*20–29*	*30–39*	*40–49*	*50–59*	*60–69*	*70–79*	*80–89*	*≥90*
**Total**, n	1865	3062	4128	3831	5690	4384	2325	384
**Smoking status**								
Almost every day	17.5	25.9	25.5	22.1	15.9	7.6	3.0	2.6
Sometimes	1.7	1.9	1.6	1.4	1.1	0.9	0.6	0.8
Past smoking	4.0	9.4	9.3	9.1	9.5	8.6	7.2	6.3
Never	76.8	62.8	63.7	67.5	73.6	82.9	89.2	90.4

* Includes smokers who were excluded from the main analysis.

Table 3 shows the results of the linear regression (involving MI) on the number of teeth (which were logarithmically transformed). In the univariate model, compared to those who were never exposed to secondhand smoke at home, those who were exposed ‘several times a week to once a month’ had a significantly higher number of teeth (β=0.09; 95% CI: 0.06–0.12). However, this association disappeared in the adjusted model; after adjusting for covariates, being exposed to secondhand smoke at home almost every day was significantly associated with having fewer teeth (β= -0.04; 95% CI: -0.07 – -0.01). Sensitivity analysis using the five categories variable of secondhand smoke exposure also showed similar results (Supplementary file Table 1).

**Table 3:** Multiple linear regression analysis of secondhand smoke at home on number of teeth* with multiple imputation (N=18812)

	*Univariate model*			*Adjusted model*		
	*β*	*95% CI*	*p*	*β*	*95% CI*	*p*
**Frequency of exposure to secondhand smoke at home**						
Almost everyday	0.02	-0.01 – 0.05	0.26	-0.04	-0.07 – -0.01	<0.01
Several times a week to once a month	0.09	0.06 – 0.12	<0.01	0.01	-0.02 – 0.03	0.64
Never (Ref.)						
**Sex**						
Men (Ref.)						
Women	0.00	3.24 – 3.31	0.64	-0.01	-0.03 – 0.01	0.30
**Age** (years)						
20 –39 (Ref. )						
40–59	-0.09	-0.09 – -0.08	<0.01	-0.09	-0.10 – -0.08	<0.01
60–79	-0.43	-0.45 – -0.42	<0.01	-0.40	-0.42 – -0.39	<0.01
≥80	-0.86	-0.89 – -0.83	<0.01	-0.78	-0.81 – -0.74	<0.01
**Occupation**						
Upper white-collar (Ref.)						
Lower white-collar	-0.05	-0.07 – -0.04	<0.01	-0.03	-0.04 – -0.01	<0.01
Blue-collar	-0.19	-0.22 – -0.17	<0.01	-0.08	-0.10 – -0.06	<0.01
Homemaker	-0.28	-0.31 – -0.26	<0.01	-0.05	-0.07 – -0.03	<0.01
Student	0.01	-0.07 – 0.08	0.85	-0.06	-0.12 – -0.01	0.03
Other	-0.54	-0.56 – -0.51	<0.01	-0.14	-0.17 – -0.11	<0.01
**Household size** (persons)						
1 (Ref.)						
2	0.12	0.09 – 0.15	<0.01	0.06	0.03 – 0.09	<0.01
3	0.23	0.20 – 0.26	<0.01	0.06	0.03 – 0.09	<0.01
≥4	0.28	0.25 – 0.31	<0.01	0.06	0.03 – 0.08	<0.01
**Dental check-up within the past year**						
Yes	0.05	0.04 – 0.07	<0.01	0.06	0.05 – 0.08	<0.01
No (Ref.)						
**Frequency of exposure to secondhand smoke outside the home**						
Almost every day	0.20	0.18 – 0.23	<0.01	-0.01	-0.04 – 0.02	0.43
Several times a week to once a month	0.21	0.19 – 0.22	<0.01	0.03	0.01 – 0.04	<0.01
Never (Ref.)						

* Logarithmic transformation was applied to number of teeth.

## DISCUSSION

The present study investigated the association between exposure to secondhand smoke at home and tooth loss among a sample of non-smokers in Japan. To the best of our knowledge, this is the first study to show, using a Japan-based nationwide survey, an association between household exposure to secondhand smoke and the number of teeth. The burden is considered to be larger among women because they showed a higher rate of exposure to secondhand smoke at home (10.9%) compared to men (3.6%).

Our multivariate analysis revealed that respondents who had frequent exposure to secondhand smoke had fewer teeth; descriptive statistics and univariate analyses failed to show this association. This inconsistency between the univariate and multivariate analysis results can be explained by negatively confounding age. As shown in Supplementary Table 1, younger people tended to smoke tobacco, and, as Table 1 shows, younger people had more teeth. Therefore, in the multivariate analysis adjusted for age, exposure to secondhand smoke was negatively associated with the number of teeth. Similar negative confounding was observed in a previous study that also examined the association between exposure to secondhand smoke and the number of teeth^13^. The study found, through univariate analysis, that older people with frequent exposure to secondhand smoke had a higher number of teeth; however, multivariate analysis adjusted for age and other confounders showed a negative association between exposure to secondhand smoke and the number of teeth. Thus, the study indicates that descriptive statistics alone cannot reveal the harmful effects of exposure to secondhand smoke because of the presence of confounding factors. Studies have found a positive association between heavy exposure to secondhand smoke at home and tooth loss. In a cohort study conducted in Australia, exposure to secondhand smoke among persons aged >45 years was associated with a significantly increased risk of tooth loss, even among non-smokers^23^. Meanwhile, in a Japan-based cross-sectional study, a significant association between exposure to secondhand smoke and tooth loss was observed among pregnant women^14^. These results are consistent with the present results.

Our results suggest that preventing secondhand smoking at home is necessary to ensuring oral health. In Japan, the implementation of non-smoking regulations has been slow compared to other countries, and secondhand smoking in workplaces and restaurants continues to represent a problem^24^. The Health Promotion Law was amended in 2018 to prohibit smoking in public places; however, there remain no regulations regarding secondhand smoking at home. Even if smokers use separate smoking areas in their homes, non-smokers living in such homes are exposed to more secondhand smoke than are nonsmokers living in non-smoking homes^25^. Therefore, it is important that smokers try as much as possible to avoid exposing their households to secondhand smoke. Additionally, knowledge of the negative effects of tobacco on health has been found to be significantly associated with reduced secondhand smoking at home^26^, hence, education regarding the negative effects of secondhand smoking is also required.

### Limitations

This study has several limitations. First, it is a cross-sectional study and therefore we could not examine the temporal relationship between tooth loss and exposure to secondhand smoke. However, the inverse association, that tooth loss increases the risk of exposure to secondhand smoke at home, seems unlikely. Second, although the self-reported number of teeth may not reflect the actual number of teeth, previous studies have reported that the self-reported number of teeth is valid when compared with the clinically examined number of teeth in Japanese adults^27,28^. Third, the reliability of the self-report data regarding secondhand smoking is uncertain because data corresponding to the exact duration of exposure to secondhand smoke were unavailable. Furthermore, the size of the rooms in a home and the number of smokers in the household also impact estimates of actual exposure to secondhand smoke. However, any misclassification of secondhand smoking and its duration would be non-differential to the report of the number of teeth. In addition, previous validation studies of self-report data regarding secondhand smoking have found that the measurements used are suitable, although self-reported exposure is generally underestimated^29^.

## CONCLUSIONS

Our analysis indicates that exposure to secondhand smoking may be associated with an increased risk of tooth loss. Further analyses controlling for other factors are also needed to confirm this finding.

## Data Availability

The data supporting this research cannot be made available for privacy or other reasons.
