# Supplementary material for: Association between exposure to secondhand smoking at home and tooth loss in Japan: A cross-sectional analysis of data from the 2016 National Health and Nutrition Survey
Source: Tob Induc Dis. 2021 Dec 9;19:96. doi: 10.18332/tid/143177 (PMC8656347; doi:10.18332/tid/143177)
Supplement: Supplementary file 1 [file TID-19-96-s1.pdf]

Supplementary Table 1. Multiple linear regression analysis of second-hand smoke at home* on number of teeth** with multiple imputation (N = 18,812).

	Univariate model					Adjusted model				
	β	[95% CI]			p	β	[95% CI]			p
Exposure to second-hand smoke at home										
Almost everyday	0.02	-0.01	to	0.05	0.23	-0.04	-0.07	to	-0.01	<0.01
Several times a week	0.10	0.05	to	0.14	<0.01	0.00	-0.05	to	0.04	0.82
once a week	0.06	0.00	to	0.11	0.04	-0.01	-0.06	to	0.04	0.73
once a month	0.11	0.06	to	0.15	<0.01	0.03	-0.01	to	0.07	0.12
Never	ref					ref				
Sex										
Men	ref					ref				
Women	0.00	-0.02	to	0.02	0.83	-0.01	-0.03	to	0.01	0.20
Age (years)										
20-39	ref					ref				
40-59	-0.09	-0.09	to	-0.08	<0.01	-0.09	-0.10	to	-0.08	<0.01
60-79	-0.43	-0.45	to	-0.42	<0.01	-0.40	-0.42	to	-0.39	<0.01
≥80	-0.86	-0.89	to	-0.83	<0.01	-0.77	-0.81	to	-0.74	<0.01
Occupation										
Upper white-collar	ref					ref				
Lower white-collar	-0.06	-0.07	to	-0.04	<0.01	-0.03	-0.04	to	-0.01	<0.01
Blue-collar	-0.19	-0.22	to	-0.17	<0.01	-0.08	-0.11	to	-0.06	<0.01
Housemaker	-0.29	-0.31	to	-0.26	<0.01	-0.05	-0.07	to	-0.03	<0.01
Students	0.02	-0.06	to	0.10	0.57	-0.06	-0.11	to	0.00	0.04
Others	-0.53	-0.56	to	-0.51	<0.01	-0.14	-0.17	to	-0.11	<0.01
The number of households										
One person	ref					ref				
Two people	0.12	0.09	to	0.15	<0.01	0.06	0.03	to	0.09	<0.01
Three people	0.23	0.20	to	0.26	<0.01	0.06	0.03	to	0.09	<0.01
Four or more people	0.28	0.25	to	0.31	<0.01	0.06	0.03	to	0.09	<0.01
Dental check-up within the past year										
Yes	0.05	0.04	to	0.07	<0.01	0.06	0.05	to	0.08	<0.01
No	ref					ref				

Exposure to second-hand smoke outside the home										
Almost everyday	0.20	0.17	to	0.23	<0.01	-0.01	-0.04	to	0.01	0.37
Several times a week to once a month	0.21	0.19	to	0.22	<0.01	0.03	0.01	to	0.04	<0.01
Never	ref					ref				

* The frequency of second-hand smoke was using the original category.

** Logarithmic transformation was applied to number of teeth.

© 2021 Inoue Y. et al.
